# Interactions of Approach and Avoidance Job Crafting and Work Engagement: A Comparison between Employees Affected and Not Affected by Organizational Changes

**DOI:** 10.3390/ijerph17239084

**Published:** 2020-12-05

**Authors:** Piia Seppälä, Lotta Harju, Jari J. Hakanen

**Affiliations:** 1 Finnish Institute of Occupational Health, Workability and Work Careers, Arinatie 3, FI-00370 Helsinki, Finland; jari.hakanen@ttl.fi; 2 EMLYON Business School, 23 Avenue Guy de Collongue, 69134 Ecully, France; harju@em-lyon.com

**Keywords:** occupational well-being, job crafting, work engagement, organizational changes, longitudinal

## Abstract

Job crafting describes proactive employee behaviors to improve the design of their work and working conditions, and to adapt their job to better suit their abilities and needs. During organizational changes, employees may use job crafting to adjust to the changes in their work and protect their well-being and motivation, i.e., work engagement. However, research shows that although the effects of job crafting strategies that expand the design of work (approach job crafting) have been positive on work engagement, the effects of job crafting strategies that diminish the scope of work (avoidance job crafting) have often been negative. This study investigated the effects of the interactions between different job crafting strategies on work engagement, an aspect that has not thus far been studied. Specifically, we hypothesized that avoidance job crafting is not harmful for work engagement when it is conducted in combination with approach job crafting, particularly during times of organizational change. A two-wave, 18-month follow-up study was conducted among public sector workers who either experienced (*n* = 479) or did not experience (*n* = 412) changes in their work. Latent moderated structural equation modeling revealed that avoidance job crafting did not reduce work engagement when combined with approach job crafting behaviors. Moreover, job crafting best benefited work engagement when it was combined with these opposing strategies. However, job crafting was beneficial for work engagement only among employees who were affected by organizational changes, that is, among employees whose job design had changed. Practically, organizations implementing changes could encourage proactive job redesign approaches among their employees—particularly both approach and avoidance types of job crafting strategies.

## 1. Introduction

Organizational changes may decrease work engagement [1,2]—an important indicator of occupational well-being [3]. This is because when employees are affected by organizational changes, they tend to perceive negative changes in their working conditions and work environment, that is, decreases in their jobs’ resourceful and motivating aspects and increases in their jobs’ demanding and hindering aspects [4,5]. Both of these have consistently shown to be detrimental to work engagement [6]. Today, organizational changes are common worldwide, and with the dawn of artificial intelligence, digitalization, robotics, Industry 4.0., and allied technologies [7,8,9,10,11,12,13,14], the future of work is changing even more rapidly, and organizational changes in work life are becoming more commonplace.

Therefore, finding effective ways to help employees adjust to changes in their work environment and sustain their well-being at work is crucial. However, traditional organizational- and management-led (top-down) approaches to work redesign may not always improve employee well-being, as they cannot cater for the various needs of all employees. Job crafting, an emerging construct in the work redesign literature, focuses on employees’ own proactive actions to shape and modify their work design [15,16,17]. It refers to self-initiated behaviors via which employees make changes to their jobs and their working conditions [18,19]. Through these proactive changes, employees may enhance their work engagement [20]—a positive state of employee well-being—[3]. Indeed, previous research has suggested that job crafting may be an especially useful strategy for dealing with organizational changes and helping employees counteract the potentially negative impacts of these changes on their work engagement [21,22]. However, although changing work environments are expected to cultivate job crafting behaviors [21,22,23,24], the current literature provides no further information on empirical studies of how job crafting relates to work engagement among employees who face organizational changes in comparison to those who do not. Thus, it is unclear whether job crafting benefits work engagement any more in changing working conditions than in a more stable work environment.

Furthermore, although the original theoretical assumption is that all job crafting activities are beneficial for work engagement [19], empirical studies have distinguished two types of job crafting that have opposite relationships with work engagement. Approach job crafting, which involves activities to expand work roles and increase the motivating aspects of a job, has shown to be beneficial for work engagement [25,26]. In contrast, avoidance job crafting, which comprises activities to reduce and avoid the demanding and hindering aspects of a job, has related negatively with work engagement [25,26]. Therefore, somewhat counterintuitively, reducing hindering job demands seems to be an unsuccessful strategy for improving work engagement, even during highly demanding work situations such as organizational changes [22]. However, empirical studies have mainly focused on investigating the unique effects of approach and avoidance job crafting activities on work engagement, as if they were mutually exclusive. Only recently have researchers started to focus on the possibility that employees may use different kinds of job crafting strategies simultaneously [27,28,29]. Indeed, a recent study found that when applied at the same time, approach job crafting may buffer the negative effects of avoidance job crafting on weekly employee functioning [28]. However, no studies have yet examined whether approach job crafting could improve the effects of avoidance job crafting on work engagement.

This study focused on these unresolved issues. Our first aim was to utilize Job Demands–Resources [30] and Conservation of Resources [31] theories to investigate whether simultaneous-approach job crafting reduces the negative effects of avoidance job crafting on work engagement. Our second aim was to examine whether employees facing changes in their work content due to organizational changes benefited from job crafting behavior more than employees not facing such changes with respect to work engagement. Therefore, this study contributes to the work engagement, job crafting, and organizational change literature by revealing whether avoidance job crafting does not harm work engagement, as was originally proposed [19], if it is utilized in combination with approach job crafting activities, and whether the organizational change context affects how job crafting enhances employees’ work engagement.

Consequently, this study offers two main practical implications for employees and organizations. First, it seeks to reveal how avoidance job crafting (decreasing stressful demands) may not be harmful work behavior in terms of well-being. In everyday work, if job crafting only had positive consequences for work engagement when employees expanded their work behaviors and roles—even during demanding and changing work situations—this would be a serious limitation for the policy recommendations of reasonable job crafting strategies. Second, to provide functional and effective practical tools for organizations, it is important to know what works best in which situations, that is, does job crafting effectively sustain work engagement during organizational changes, and could it then be even more effective than during more stable times?

### 1.1. Conceptualization of Job Crafting and Work Engagement

The present study utilized Job Demands and Resources theory (JD-R theory) [30] to frame and operationalize the study constructs. JD-R theory is a comprehensive job characteristics theory that aims to explain well-being and motivation at work. According to its framework, well-being and motivation at work are a function of the motivating (such as social support) and the demanding (such as emotionally demanding clients) conditions or aspects of a job. However, in contrast to the more traditional job characteristics theories [32], which see managers as being responsible for job (re)design [15,16,17], JD-R theory assumes that employees can themselves make self-initiated and future-directed changes to their job design and balance motivating and demanding job conditions in order to promote their well-being and motivation [30]. Within the JD-R framework, this proactive behavior is coined job crafting [19].

JD-R theory defines job crafting as a multidimensional construct consisting of four sub-dimensions that capture different ways in which employees may craft their jobs [33]. Three of the dimensions focus on expanding one’s job, that is, increasing structural job resources (such as work activities to develop one’s capacities or learn new skills at work), increasing social job resources (such as activities to seek social support and advice from a supervisor), and increasing challenging job demands (such as activities to voluntarily increase responsibilities at work or begin new self-initiated projects at work). One of the dimensions focuses on contracting the job, that is, decreasing hindering job demands (such as activities to avoid emotionally straining job situations or aiming to make work mentally less demanding) [33]. Thus, job crafting consists of two distinct and opposite strategies via which employees can modify their job conditions and characteristic—increasing job resources and challenging job demands, and decreasing hindering job demands. These strategies have been called approach and avoidance job crafting [25,34,35].

Work engagement is defined as an affective motivational and positive psychological state in which employees are vigorous, dedicated to, and absorbed in their work [3]. Thus, when employees are engaged at work, they feel energetic and willing to invest effort into their work (vigor); they are committed to and involved in their work (dedication); and they are often so focused on their work that they even lose their sense of time (absorption). Work engagement is further associated with several outcomes desired by organizations and individual employees [36,37], such as high performance [38] and organizational commitment [39], as well as life satisfaction [40] and recovery after work [41]. Work engagement may also be especially useful during organizational change processes: studies have found that the more engaged employees are, the more open they are to organizational changes, the better they adapt to them, and the more organizational citizenship behaviors and fewer deviant behaviors they show [42,43].

### 1.2. Relationship between Approach and Avoidance Job Crafting and Work Engagement

According to JD-R theory, approach job crafting behaviors that aim to increase the resourceful and stimulating aspects of a job (that is, increasing structural and social job resources and challenging job demands), motivate, and enable development at work, which engages employees in their jobs [30]. In contrast, avoidance job crafting behaviors to decrease the constraining and demanding aspects of a job reduce the cognitive and emotional requirements of a job, which in turn hinder work engagement. Thus, these different types of job crafting behaviors enable an employee to balance the resourceful and demanding aspects of a job and enhance work engagement [19,30]. Previous empirical studies have supported the theoretical expectation that approach job crafting behaviors are positively related to work engagement [20,44,45,46,47,48,49]. However, in contrast to original theoretical expectations [19], avoidance job crafting has not always been associated with work engagement [50]; it has even been negatively related to work engagement [22,51,52]. The motivating and disengaging natures of separate job crafting strategies might explain this unexpected finding.

Increasing job resources and challenging job demands (approach job crafting) is expected to stimulate and activate individuals by facilitating the accomplishment of work-related goals and promoting personal growth and development at work [30]. These are further expected to trigger a positive motivational process, which leads to work engagement. In contrast, by decreasing hindering job demands (avoidance job crafting), employees aim to reduce the excessive cognitive and emotional requirements of a job to avoid strain. However, as avoidance job crafting orients a person away from one’s job, it may also reduce the motivating and important parts of work and impoverish the design of a job [53]. Avoidance job crafting in the form of reducing hindering job demands may help individuals cope with stressors but not motivate them further [30].

We also used the Conservation of Resources theory (COR) [31,54] to explore the effects of approach and avoidance job crafting on work engagement. COR theory is a resource-based stress theory that explains individual behavior based on the motivation to acquire and conserve resources. It is also a widely used theory in work engagement studies. Its main assumption is that individuals are motivated to maintain and accumulate the resources that they value (such as well-being and health). COR theory posits that individuals experience ill-being and stress when they are faced with resource threats or the loss of important resources [54]. COR theory distinguishes between two different kinds of behaviors via which individuals may maintain and accumulate their valued resources. First, proactive coping, which refers to behaviors that aim to attain and acquire new resources, and second, reactive coping, which refers to behaviors that aim to reduce stressful demands to protect existing resources [31]. This distinction closely aligns with the approach and avoidance job crafting strategies in the job crafting literature [25,34,35].

COR theory further notes that in order to acquire new resources a person must use their existing resources (e.g., time, energy). However, the risk is that they may not attain their desired resources, and that in the process of attempting to attain them, they might sacrifice, i.e., lose the resources they already had [31]. Thus, employees may rather try to protect their resources by diminishing the demanding aspects of their job. However, decreasing demands requires resources such as time and energy, but demands are not usually easy to alter, at least in the short term [55]. Therefore, reducing demands may only deplete an individuals’ resources, and they may not actually gain anything [28]. Consequently, if employees focus solely on decreasing hindering job demands, they may deplete their existing resources, which reduces work engagement.

COR theory also posits that although proactive and reactive coping behaviors are distinguishable, they are often carried out hand in hand, which is expected to have beneficial consequences for individuals’ well-being [31]. Thus, individuals tend to react to demands and aim to diminish them but also actively mobilize and build new resources. By building new resources, individuals may mitigate the negative effects of losing the resources allocated to diminish demands [56]. These propositions have gained support in empirical coping studies, which have shown that individuals tend to use both proactive and reactive ways of coping together and that one strategy supplements the other [57,58]. Previous studies have also found that the use of reactive coping behavior alone is not beneficial for well-being [59,60], but that it may lead to positive emotions if it is used simultaneously with proactive coping behavior [57,60]. Thus, the potential well-being benefits of reactive coping may depend on the simultaneous use of proactive coping behavior.

Furthermore, recent empirical job crafting studies have suggested that the effects of avoidance job crafting on employee and organizational outcomes may be positive if employees simultaneously carry out approach job crafting [27,28,29]. Mäkikangas [27] found in a day-level study utilizing a person-centered research approach that employees tended to utilize approach and avoidance job crafting strategies together, which was further related to higher daily work engagement. In addition, Petrou and Xanthopoulou [28] found that approach job crafting strategies buffered the negative relationship between avoidance job crafting and employability. They also found that avoidance job crafting, together with approach job crafting, boosted weekly employee performance.

Therefore, sometimes, such as during demanding organizational changes, an employee may need to decrease hindering job demands to make their work less intense. However, to remain engaged in their work, it is also important that they build the motivating and resourceful aspects of their jobs. For example, when employees seek advice from their supervisors (i.e., increasing social job resources) on, for instance, which work tasks to prioritize when their workload is heavy, this may counteract the possible negative disengaging effects on work engagement resulting from avoiding some existing work tasks. Furthermore, voluntarily learning new things at work and developing their capabilities (increasing structural job resources) or actively confronting new challenges (increasing challenging job demands) may provide the employee with an opportunity to gain new resources and compensate for the loss of the resources they invested in diminishing some aspects of their job. Based on the theorizing above [31,54] and on recent research findings [27,28], we hypothesize that approach job crafting reduces the negative effects of avoidance job crafting on work engagement.

**Hypothesis** **1.** *Approach job crafting (i.e., increasing structural job resources, increasing social job resources, and increasing challenging job demands) at T1 moderates the relationship between avoidance job crafting (i.e., decreasing hindering job demands) at T1 and work engagement at T2. The relationship between avoidance job crafting at T1 and work engagement at T2 is not negative when approach job crafting is higher (vs. lower)*.

Finally, COR theory also posits a gain paradox principle, which states that protecting and attaining valued resources becomes especially important for well-being when resources are threatened [56], such as during demanding organizational changes. Paradoxically, fostering and building resources improves their value in demanding contexts, that is, in a context in which employees may focus more on protecting their resources [61]. Indeed, it has been suggested that job crafting behavior is particularly useful for work engagement during demanding organizational changes [21,22,23,62], but thus far, no studies have explicitly tested this. To contribute to the existing knowledge, we tested the study hypotheses among employees facing changes in their work content because of organizational changes and among employees who were not facing changes in their work content. In this way, it was possible to gain deeper insights into the relationship between job crafting and work engagement in a context in which employees experience a great deal of changes compared to a context in which they do not, and to determine whether employees undergoing changes benefit more from job crafting in terms of future work engagement. Although job crafting is a self-initiated activity that focuses on improvements to one’s own job, the job situation likely triggers the motivation and need to craft one’s job [18,24]. Thus, the longitudinal effects of job crafting may not be independent from the organizational context in which they are performed.

Based on the assumptions of COR theory [31,56] and on previous suggestions [22,43], we hypothesize that job crafting behavior has more impact on work engagement when employees are dealing with organizational changes.

**Hypothesis** **2.** *The relationships between job crafting behaviors and work engagement are stronger among employees facing changes in their work contents than among employees not facing such changes.*

## 2. Materials and Methods

### 2.1. Participants

This two-wave follow-up study utilized a dataset that was collected as part of a larger longitudinal research project consisting of several sub-projects that examined the impact of the changes brought about by the regional government and health and social services reform (i.e., reforms targeting municipalities and reforms implemented by the municipalities themselves) in 34 municipalities in Finland (2016–2018). The policymakers of the municipal organizations expected the research project to provide practical and useful information on how to help workplaces maintain employees’ well-being in the face of current but also possible future changes. These municipal reforms primarily included organizational restructures such as municipal mergers, which caused the reorganization of employees’ job descriptions and work content. The reorganization involved no personnel dismissals, and the aim of the changes was to improve the functions of the municipal organizations. The selection of two measurement points and 18 months as the follow-up period was based on practical decisions by the Association of Finnish Local and Regional Local Authorities. The research project followed the informed consent procedure as well as the ethical principles of the responsible conduct of research. The study protocol was approved by the Ethics Board of the Finnish Institute of Occupational Health (Hakanen 01 16; 29.1.2016).

Participants responded to the electronic questionnaire twice: in 2016 (Time 1, T1) and 18 months later in 2017 (Time 2, T2). At T1, the electronic questionnaire was sent to a total of 84,600 employees, of whom 10,920 responded. Furthermore, at T2, the questionnaire was sent to the 4302 employees who at T1 had indicated that they wished to participate in the follow-up study. Of the 4302 participants, 2453 responded at both measurement times (response rate 57%). The present study focused on the participants who responded to the questionnaire at both time points. However, the organizational changes took place at different phases; the different municipalities and their units did not undergo them simultaneously. As the aim of the study was to compare the effects of job crafting among employees facing changes with the effects among those who were not facing such changes, the study focused on the participants who reported either having experienced many changes in their work content at T1 or not having experienced any changes in their work content at T1. Altogether 479 participants reported having experienced either many or very many changes in their work content, and 412 participants reported that they had experienced no changes in their work content (elicited by asking “Have you experienced changes in your work content recently?”).

Of the participants who reported experiencing many changes (*n* = 479), most were female (86%). The mean age at the first measurement time was 48.8 years (SD = 9.65). Nearly all (90%) were permanently employed and the vast majority (80%) worked in a non-supervisory position. Most of the participants who reported no changes in their work content (*n* = 412) were female (82%). The mean age at the first measurement point was 50.0 years (SD = 9.36). Nearly all (91%) were permanently employed, and the majority (80%) worked in a non-supervisory position. Both groups of participants worked in various industrial fields and represented several occupations such as education, day care, library services, construction supervision, land use planning, road and street maintenance, public transport services, technical services, waste management, and social and health services. The groups worked in different parts of Finland.

The comparisons between the participants facing changes and those who were not facing changes at T1 revealed that those facing no changes were slightly older (F(1, 878) = 11.63, *p* = 0.001) than those facing changes. Furthermore, the participants facing changes reported increasing structural job resources (F(1, 889) = 10.58, *p* = 0.001) and increasing challenging job demands somewhat more often (F(1, 888) = 7.83, *p* = 0.005). The participants did not differ in terms of any other demographics or study variables.

### 2.2. Measures

#### 2.2.1. Job Crafting

Job crafting was measured using the Job Crafting Scale [33], which is a well-validated, widely utilized questionnaire for studying job crafting, also in Finnish organizations [33,63,64]. In this study, we used a shortened version of the scale to prevent response fatigue. All four sub-scales: increasing structural job resources, increasing social job resources, increasing challenging job demands, and decreasing hindering job demands were measured using three items instead of the original five/six. The scale was shortened on the basis of both the face validity of the items and confirmatory factor analysis (CFA), using a previously collected dataset in the same country (*n* = 11,468 employees representing a variety of professions and workplaces) [64]. Increasing structural job resources were assessed using the following items: “I try to develop my capabilities; I try to develop myself professionally; and I try to learn new things at work” (for employees facing changes T1 α = 0.93; for employees not facing changes T1 α = 0.93), and increasing social job resources were assessed using items: “I ask my supervisor to coach me; I ask whether my supervisor is satisfied with my work; and I look to my supervisor for inspiration” (for employees facing changes T1 α = 0.83; for employees not facing changes T1 α = 0.83). Increasing challenging job demands were assessed using the following items: “When an interesting project comes along, I offer myself proactively as project co-worker; When there is not much to do at work, I see it as a change to start new projects; and I try to make my work more challenging by examining the underlying relationship between aspects of my job” (for employees facing changes T1 α = 0.70; for employees not facing changes T1 α = 0.67). Finally, decreasing hindering job demands were assessed using items: “I make sure that my work is mentally less intense; I try to make sure that my work is emotionally less intense; I organize my work so as to minimize contact with people whose expectations are unrealistic” (for employees facing changes T1 α = 0.72; for employees not facing changes T1 α = 0.69). The items were rated on a five-point scale from 1 (totally disagree) to 5 (totally agree). In the previous validation study, Cronbach’s alpha was 0.82 for increasing structural job resources, 0.77 for increasing social job resources, 0.75 for increasing challenging job demands, and 0.79 for decreasing hindering job demands [33].

#### 2.2.2. Work Engagement

Work engagement was assessed using the short version of the Utrecht Work Engagement Scale (UWES) [65], which is the most widely utilized scale for measuring work engagement and is also well-validated in Finnish samples [66]. All three sub-scales were measured using three items. Vigor was assessed using items such as “At my work, I feel bursting with energy” (for employees facing changes T1 α = 0.87, T2 α = 0.88; for employees not facing changes T1 α = 0.85, T2 α = 0.86), dedication was measured using items such as “I am enthusiastic about my job” (for employees facing changes T1 α = 0.88, T2 α = 0.91; for employees not facing changes T1 α = 0.88, T2 α = 0.90), and absorption was assessed using items such as “I feel happy when I am working intensely” (for employees facing changes T1 α = 0.82, T2 α = 0.83; for employees not facing changes T1 α = 0.84, T2 α = 0.86). The items were ranked on a seven-point rating scale (0 = never to 6 = every day). The mean of the total scores for the three dimensions of work engagement were calculated separately as the mean of the corresponding three items, and the total scores were used as indicators of a latent work engagement factor in subsequent analyses. Cronbach’s alpha in the previous validation study varied from 0.81 to 0.85 for vigor, from 0.83 to 0.87 for dedication, and from 0.75 to 0.83 for absorption [66].

### 2.3. Statistical Analyses

#### 2.3.1. Confirmatory Factor Analysis of Job Crafting

We first utilized CFA in a structural equation modeling (SEM) framework to investigate the distinctiveness of the four job crafting behaviors. Thus, we tested whether the four-factor structure (i.e., increasing structural job resources, increasing social job resources, increasing challenging job demands, and decreasing hindering job demands) would fit the dataset. As previous studies have also supported other factor structures [62,67], we tested alternative models: a three-factor model (in which structural and social job resources were loaded on the same factor), and a two-factor model (in which increasing structural job resources, increasing social job resources, and increasing challenging job demands were loaded on the same factor). All analyses were conducted using the Mplus statistical package (version 7.4) (Muthén & Muthén, Los Angeles, CA) [68]. The conventional cut-off values of the fit indices were used [69,70,71]. For the root mean square error of approximation (RMSEA), the cut-off points ranged between 0.05 and 0.10. For both the Comparative Fit Index (CFI) and the Tucker–Lewis Index (TLI), values above 0.90 indicated an acceptable fit. For standardized root mean squared residual (SRMR), well-fitting models obtained values of less than 0.05, and values of less than 0.08 are deemed acceptable

The CFA showed that the fit of the four-factor model was good (RMSEA = 0.052; CFI = 0.972; TLI = 0.961; SRMR = 0.043) among the employees facing changes, whereas the covariance matrix of the three-factor model or the two-factor model was not positively definite, indicating that these models were inadmissible. Thus, they were excluded from the subsequent analyses. The results among the employees not facing changes were similar and showed that the fit of the four-factor model was good (RMSEA = 0.044; CFI = 0.978; TLI = 0.970; SRMR = 0.051), but the covariance matrix of the three-factor model or the two-factor model was not positively definite. All the factor loadings of the four-factor model were significant and loaded on their respective factors (among both groups). However, increasing structural job resources and increasing challenging job demand factors correlated strongly with each other (*r* = 0.612 among employees not facing changes and *r* = 0.729 among employees facing changes). Consequently, to avoid multicollinearity problems [72], approach job crafting factors were included in the further models one at a time. This enabled us to examine whether some of the approach job crafting strategies were more effective than others in buffering the negative effects of avoidance job crafting on work engagement.

#### 2.3.2. Hypothesis Testing

A latent moderated SEM framework (LMS) was used to test the hypotheses [73,74]. By using a latent variable framework, it was possible to produce estimates of interactions that were unattenuated by measurement error, and this increased the study’s power and reduced the likelihood of biased estimates. The full information maximum likelihood estimation (FIML) method enabled the use of all the information in the dataset to estimate the parameters in the models without imputing data.

**Figure 1:**
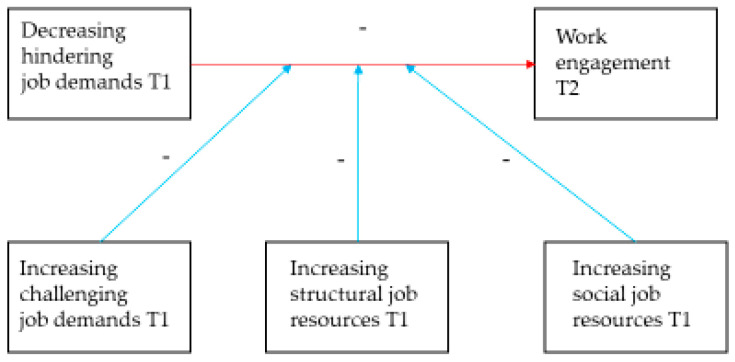
Investigated research model. Note. T1 = Time 1; T2 = Time 2, 18 months after T1.

The models were tested in two main steps, separately for each group of employees. First, a model without the latent interaction term was estimated, i.e., a model in which the main effects of each of the approach job crafting factors (i.e., increasing structural or social job resources, or challenging job demands) and avoidance job crafting factor (i.e., decreasing hindering job demands) on work engagement were estimated (i.e., a baseline model, Model 0; M0). The fit of the baseline model was evaluated using RMSEA, TLI, CFI, and SRMR indices (for cut-off values, see Section 2.3.1). Second, we tested a model in which the main effects and the latent interaction term (i.e., interaction between the approach job crafting factors and avoidance job crafting factors) were estimated (i.e., an alternative model, Model 1, M1). Figure 1 presents the investigated research model.

An interaction effect becomes evident when the regression coefficient (β-coefficient) from the interaction variable to the outcome variable (i.e., work engagement) is statistically significant. However, to further support the significance of an interaction term, the baseline model was compared to the alternative model. The fit of the two models was compared using the log-likelihood ratio test value (D), which is approximately distributed as χ^2^ [73]. Finally, a graphical presentation was used to interpret the interaction effects [75]. To do this, in cases of significant interactions, the standardized values of work engagement under conditions of one standard deviation above (high) and below (low) the mean of the three approach job crafting factors, and one standard deviation above (high) and below (low) the mean of the decreasing hindering job demands factor were calculated. The predicted relationships (i.e., the simple slopes) between decreasing hindering job demands and work engagement at different levels of increasing job resources and/or challenging job demands were then graphically presented.

## 3. Results

### 3.1. Descriptive Statistics

Table 1 and Table 2 present the means, standard deviations, and correlations of the study variables. Increasing structural and social job resources and increasing challenging job demands were positively related to work engagement, and decreasing hindering job demands was negatively related to work engagement in both groups of employees.

### 3.2. Hypothesis-Testing among Employees Facing Changes in Their Work Content

First, all the M0 models showed a reasonable fit with the dataset (RMSEA = 0.07–0.08; CFI = 0.94–0.95; TLI = 0.91–0.93; SRMR = 0.049–0.052) among the employees facing changes. However, after controlling for the level of work engagement at T1, the direct associations between job crafting factors at T1 and work engagement factor at T2 were non-significant. The baseline level of work engagement explained 42–48% of the variance of work engagement at T2. Table 3 presents the results of the LMS.

Second, the results showed that the interaction term of increasing challenging job demands and decreasing hindering job demands (β = 0.139, *p* = 0.003) at T1, and the interaction term of increasing social job resources and decreasing hindering job demands (β = 0.106, *p* = 0.012) at T1 for work engagement at T2 were positive and significant (see Table 3). In contrast, the interaction term of increasing structural job resources and decreasing hindering job demands at T1 was not significant for work engagement at T2 after controlling for work engagement at T1 (β = −0.007, *p* = 0.883). Consequently, the log-likelihood ratio tests showed that two out of three M0 models showed a poorer fit than the M1 models (see Table 3).

Next, we calculated the predicted values of work engagement under conditions of ±1 SD from the mean of increasing challenging job demands and decreasing hindering job demands, and under conditions of ±1 SD from the mean of increasing social job resources and decreasing hindering job demands. Table 4 summarizes the results of the simple slopes. It shows that the effect of high decreasing hindering job demands on work engagement was negative, when increasing challenging job demands was low (β = −0.414, *p* = 0.016). In contrast, the effect of decreasing hindering job demands on work engagement was not negative when increasing challenging job demands was high (β = 0.212, *p* = 0.030). Indeed, the effect on work engagement of decreasing hindering job demands in combination with increasing challenging job demands was positive, and thus, this combination boosted future work engagement even further. In addition, the effect of decreasing hindering job demands on work engagement was negative when increasing social job resources was low (β = −0.331, *p* = 0.021), whereas this negative effect was reduced when increasing social job resources was high, making it no longer harmful to work engagement (β = 0.191, *p* = 0.062). Thus, the simultaneous use of increasing social job resources buffered the negative effect of decreasing hindering job demands on work engagement. Consequently, H1 was partially supported, as increasing challenging job demands and increasing social job resources, but not increasing structural job resources, moderated the relationship between decreasing hindering job demands and work engagement in such a way that it was not harmful when approach job crafting behavior was higher (vs. lower).

**Figure 2:**
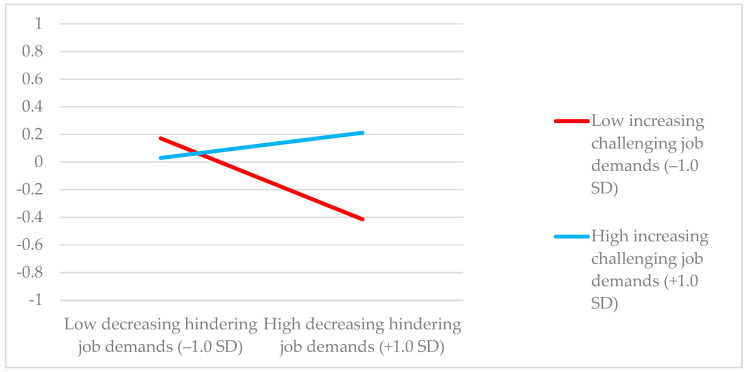
Interaction effect of increasing challenging job demands and decreasing hindering job demands at T1 on work engagement at T2.

**Figure 3:**
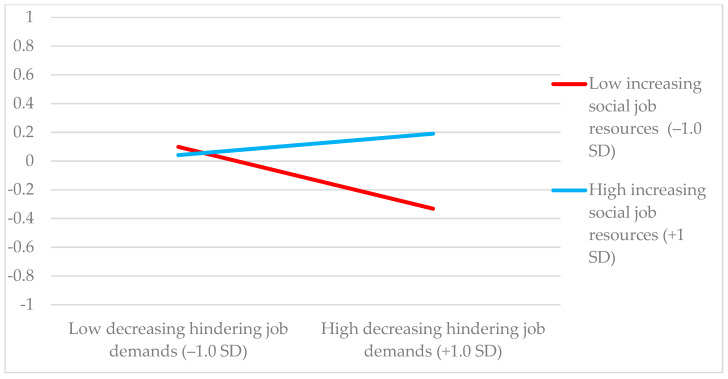
Interaction effect of increasing social job resources and decreasing hindering job demands at T1 on work engagement at T2.

Figure 2 and Figure 3 illustrate the relationship between decreasing hindering job demands and work engagement when levels of increasing challenging job demands and increasing social job resources are high and low.

### 3.3. Hypothesis-Testing among Employees Not Facing Changes in Their Work Content

All the M0 models showed a reasonable fit with the dataset (RMSEA = 0.09–0.10; CFI = 0.90–0.93; TLI = 0.86–0.90; SRMR = 0.050–0.051). After controlling for the level of work engagement at T1, the direct associations between job crafting factors at T1 and work engagement factors at T2 were non-significant (see Table 5). The stability of work engagement was very high, and the baseline level of work engagement explained 62–65% of the variance of work engagement at T2. Furthermore, in contrast to H1, none of the interactions between approach job crafting factors and avoidance job crafting factor were significant among the participants not facing changes in their work content. Consequently, as this study found no longitudinal relations between job crafting and work engagement (neither direct nor interaction effects) among employees not facing changes in their work content, H2—that the relationships between job crafting and work engagement would be stronger among employees facing changes than among employees not facing changes—was partially supported.

Finally, supplementary analyses were conducted to test the study hypotheses among all the employees in the dataset (*n* = 2453, please see Appendix A for details and results).

## 4. Discussion

The aim of this two-wave follow-up study with an 18-month interval was to utilize the theoretical propositions of JD-R [30] and COR theories [31,56] to test whether approach job crafting reduces the harmful effects of avoidance job crafting on future work engagement, particularly among employees facing changes in their work content because of organizational changes. Our findings suggest that the potentially negative impact of avoidance job crafting on work engagement may be buffered by simultaneous approach job crafting. Indeed, job crafting seems to work best as a combination of these different types of proactive behaviors, which jointly enhance well-being, but only among those facing changes. These main study findings are discussed in more detail below.

### 4.1. Combined Approach and Avoidance Job Crafting Behaviors Benefit Work Engagement during Organizational Changes

First, among the group of employees who faced many changes in their work content, we observed an interaction between two forms of approach job crafting behaviors and avoidance job crafting. More specifically, as expected, increasing social job resources buffered the detrimental effects of reducing hindering job demands on work engagement, and avoidance job crafting lost its harmful effects when increasing social job resources was higher. In addition, when employees engaged in decreasing hindering job demands and increasing challenging job demands in tandem, their work engagement was not only maintained, but it improved. Therefore, whereas increasing social job resources had a buffering effect, increasing challenging job demands had a boosting effect. An explanation for the different interaction effects may be that by receiving support and guidance an employee may buffer the negative effects on work engagement of avoiding some work tasks. An employee may, for example, obtain advice to focus on important tasks and pay less attention to irrelevant tasks. However, although increasing social resources combined with decreasing hindering job demands protect well-being from the negative effects of avoidance behavior, this strategy does not motivate employees any further. Thus, to truly increase work engagement in changing circumstances, an employee may need to withdraw from some work tasks or roles and simultaneously take up new challenges and seek new responsibilities. These findings are in line with a recent study that also found different interaction patters between various approach job crafting strategies and avoidance job crafting [28].

One approach type of job crafting—increasing structural job resources—showed no interaction effects on work engagement when combined with avoidance job crafting. One explanation for this non-significant finding could be that as the employees were facing changes that primarily affected their work content, increasing structural job resources, which involves behaviors intended to alter a person’s job resources at the level of current work tasks [19], may not have been considered appropriate behavior in the long term. It is also possible that this non-significant result reflects a measurement issue, that is, social desirability (as who would disagree with trying to develop capabilities?), which may have caused a ceiling effect (the mean of increasing structural job resources was 4.3 on a scale of 1–5). The lack of variability may then have attenuated the relationship with decreasing hindering job demands. Nevertheless, this finding is in line with two previous studies that have found that approach job crafting in general does not always buffer the negative effects of avoidance job crafting on supervisor support [29], and that not all approach job crafting strategies are equally effective in reducing the negative effects of avoidance job crafting on employee functioning [28]. As this study was a pioneer in examining the possible moderating effects of approach job crafting on the relationship between avoidance job crafting and work engagement, future studies could further clarify which approach job crafting strategies work best for this purpose.

Furthermore, we found no longitudinal main effects, and none of the job crafting types affected work engagement as a single strategy. In the context of change, neither approach nor avoidance crafting strategies alone were enough, but the use of these strategies together benefited work engagement. Thus, in situations in which they faced concrete changes in their work, it seems that employees needed to use different types of job crafting strategies (i.e., approach and avoidance job crafting) in order to sustain and foster their work engagement.

Second, we found no longitudinal interaction or main effects among the group of employees not facing changes in their work content. On the one hand, this result may resonate with the rather long timeframe of this study (18 months). Previous studies have shown that adapting to changes and crafting a job during organizational changes may take time [76], whereas a work situation without these changes may not stimulate a strong need to craft a job, at least in the long term, and the consequences of job crafting might be shorter lived. Thus, this study adds to the existing knowledge regarding the long-term effects of job crafting, as longitudinal studies on the effects of job crafting behaviors on work engagement in general are still scarce [20]. On the other hand, this result aligns with the proposition that although job crafting is a self-initiated activity that focuses on an individual’s own job, the motivation and need to craft one’s job is triggered in situations that require it [18,24]. Thus, partially as expected, the relationships between job crafting and work engagement were stronger among the employees facing changes in their work content than among employees not facing these changes. All in all, our study suggests that the effects of job crafting on work engagement may not be independent from the organizational situation.

### 4.2. Theoretical and Practical Implications

This study makes three main contributions to the literature. First, although the recent conceptual overviews of job crafting [34] have suggested that avoidance job crafting may not always necessarily have negative consequences, empirical studies showing how and when avoidance job crafting may not reduce work engagement are still sparse [77]. This study showed that the effect of avoidance job crafting on work engagement depended on the simultaneous utilization of some types of approach job crafting strategies: the harmful effects of avoidance job crafting on work engagement could be counteracted by simultaneous approach job crafting behavior. Second, this study revealed that neither approach nor avoidance job crafting affected work engagement in the long term as an independent strategy, but the combined utilization of these different strategies benefited work engagement. Thus, following the recent line of research [27], this study suggests that job crafting seems to work best when different types of proactive behaviors are jointly utilized. This is in line with the assumptions of COR theory [31]: that individuals need to both decrease demands and actively mobilize new resources to foster well-being, and it is the combination of these different kinds of behaviors that jointly benefits work engagement.

Third, this study contributed to the literature by investigating the contextual factors that may play an important role in how job crafting affects work engagement. This study showed that job crafting did not promote the work engagement of all employees in the same way. In contrast to the employees not facing changes, the employees facing changes benefited from a combination of approach and avoidance job crafting strategies in terms of future work engagement. Therefore, this study revealed that the impact of job crafting activities is not independent of the organizational context in which they are performed; instead the contextual factors seem to be salient in explaining the longitudinal impacts of job crafting [24,78]. Consequently, in line with the COR theory’s assumption that protecting and fostering resources becomes especially important in demanding contexts [56], this study showed that approach and avoidance job crafting benefited work engagement among the employees whose jobs had changed—that is in the demanding context that required it. 

The study findings also provide new practical insights for organizations implementing changes. Employees today face very different kinds of changes in their work [7,8,9,10,11,12,13,14], some of which are highly unpredictable, such as the sudden change from working at workplaces to remote work due to the current coronavirus pandemic. Thus, for many reasons, current work life is causing growing needs for proactive and self-initiated ways to adapt to these changes and sustain well-being. This study suggests that job crafting can indeed be an effective and valuable tool for maintaining and even enhancing work engagement when facing changes. A vast amount of previous research has shown that job crafting behavior can be learnt, and many job crafting interventions have been conducted in recent years [79,80]. However, because of the previous indications of the negative effects of avoidance job crafting, interventions have sometimes focused more on optimizing hindering demands, such as aiming to work more efficiently [81], than decreasing them, and some studies have excluded avoidance job crafting strategy altogether from the content of their interventions [82,83]. This study recommends using intervention exercises and practices to stimulate employees to use not only approach job crafting activities, but also avoidance job crafting strategies. That is, to increase motivating and energizing challenges and job resources, but also to decrease the straining demands of a job, particularly during demanding times such as organizational changes. This evidence-based knowledge on effective practices is needed, as job resources tend to decrease and job demands to increase during organizational changes [4,5].

However, it is noteworthy that in practice, successfully decreasing some kinds of hindering job demands by self-initially crafting one’s job may be difficult or sometimes even impossible [55]. For example, proactive actions to avoid interacting with negatively behaving clients or to reduce physical job demands may not always be feasible. Therefore, although we propose that job crafting is a useful tool for improving working conditions, it is not necessarily always suitable or sufficient for all types of job redesigns in organizations. Organizational policies and management also play an important role in changing and improving the design of a job, especially in managing and controlling the amount of hindering demands of a job. Thus, combining proactive bottom-up employee-led and strategic top-down management-led job redesign approaches may lead to the best possible outcomes [84].

### 4.3. Study Limitations and Suggestions for Future Studies

A few limitations of this study require consideration. First, the generalizability of the sample needs to be considered carefully. As the majority (80%) of municipal sector workers in Finland are female [85], the sample of this study was also female dominated. Thus, although the sample was not balanced with respect to gender, it was correctly representative of the gender distribution among Finish municipal workers. A previous meta-analysis found that women tend to report higher levels of job crafting than men [26]. The differences found in the meta-analysis were, however, small, and job crafting theory does not make any specific gender assumptions [19,30]. Furthermore, this study focused on public sector workers only. However, occupation has been shown to be much more important for work engagement than sector [86], and the current study consisted of many different occupational groups. Still, the relationships found are mostly generalizable to female public sector workers. Second, some features of the study design need to be acknowledged. Because of the rather long timeframe of this study, it is possible that some changes other than those to work content may have occurred and encouraged employees to craft their jobs. Furthermore, because of the long time-lag, possible temporary consequences of job crafting may have been missed. However, most previous job crafting studies have utilized cross-sectional datasets or follow-up studies with short time-lags in the contexts of organizational changes [23,50,62], and longer-term individual consequences of job crafting have recently been called for [87].

Third, related to measurement issues, as the original job crafting scale includes 21 items, the short version of the scale was utilized in order to reduce burden and motivate the participants to reply. Although we consider that the selected 12 items sufficiently captured the phenomenon being measured [64], the results may not be fully comparable with those of previous studies that have used the original job crafting scale. Therefore, future studies could replicate the findings of this study using the original operationalization of the job crafting measure. Fourth, and finally, common method variance bias may emerge when study constructs are measured using self-reports. However, the use of a longitudinal dataset and interaction effects reduces this risk [88,89]. Future studies could still combine different methods, such as self-reports and peer-reports when investigating job crafting and work engagement. They could also consider multidisciplinary methods for revealing the consequences of job crafting. For example, future studies could examine whether avoidance job crafting, as the sole job crafting strategy, would relate differently to various psychophysiological correlates than if used jointly with approach crafting (i.e., does decreasing hindering job demands yield to less psychophysiological costs as assumed, or should it be combined with increasing resources?).

## 5. Conclusions

This study showed that the negative effects of avoidance job crafting (in the form of decreasing hindering job demands) on future work engagement can be reduced by simultaneously using approach job crafting strategies (in the form of increasing job resources and challenges). Neither approach nor avoidance job crafting strategies alone predicted future work engagement; only the use of these strategies together was effective. Furthermore, long-term benefits depended on the context in which these job crafting strategies were employed: job crafting was an effective strategy among employees facing changes. Thus, this study showed how avoidance job crafting is not harmful to work engagement (combining it with approach crafting behaviors), and when job crafting is especially useful and promotes work engagement in the longer term (during changes). Consequently, to effectively deal with the changes and to keep employees engaged, we recommend that organizations implementing changes provide employees with opportunities to decrease excessive demands and simultaneously increase resources. To further boost work engagement in these situations, employees need opportunities to decrease excessive demands in tandem with increasing new challenges.

## Figures and Tables

**Table 1:** Means, standard deviations, and Pearson correlations of study variables among participants facing changes in their work content (*n* = 479).

Variables	M	SD	1.	2.	3.	4.	5.
1. Increasing structural job resources T1	4.32	0.65					
2. Increasing social job resources T1	2.41	0.89	0.33 ***				
3. Increasing challenging job demands T1	3.73	0.73	0.62 ***	0.35 ***			
4. Decreasing hindering job demands T1	2.20	0.78	−0.04	0.07	−0.05		
5. Work engagement T1	4.91	1.07	0.39 ***	0.29 ***	0.41 ***	−0.24 ***	
6. Work engagement T2	4.72	1.21	0.23 ***	0.23 ***	0.31 ***	−0.24 ***	0.64 ***

Note. * *p* < 0.05, ** *p* < 0.01, *** *p* < 0.001. T1 = Time 1; T2 = Time 2, 18 months after T1. 1 = Increasing structural job resources T1; 2 = Increasing social job resources T1; 3 = Increasing challenging job demands T1; 4 = Decreasing hindering job demands T1; 5 = Work engagement T1; 6 = Work engagement T2.

**Table 2:** Means, standard deviations, and Pearson correlations of study variables among participants not facing changes in work content (*n* = 412).

Variables	M	SD	1.	2.	3.	4.	5.
1. Increasing structural job resources T1	4.18	0.69					
2. Increasing social job resources T1	2.30	0.87	0.22 ***				
3. Increasing challenging job demands T1	3.59	0.73	0.49 ***	0.29 ***			
4. Decreasing hindering job demands T1	2.22	0.80	−0.13 **	0.02	−0.11 *		
5. Work engagement T1	4.97	1.05	0.30 ***	0.24 ***	0.30 ***	−0.26 ***	
6. Work engagement T2	4.71	1.25	0.22 ***	0.22 ***	0.23 ***	−0.21 ***	0.72 ***

Note. *** *p* < 0.001. T1 = Time 1; T2 = Time 2, 18 months after T1. 1 = Increasing structural job resources T1; 2 = Increasing social job resources T1; 3 = Increasing challenging job demands T1; 4 = Decreasing hindering job demands T1; 5 = Work engagement T1; 6 = Work engagement T2.

**Table 3:** Results of LMS: interaction effects of job crafting dimensions on work engagement among participants facing changes in their work content (*n* = 479).

T1 Variables	M0 T2 Work Engagement β (SE)	*p*	M1 T2 Work Engagement β (SE)	*p*
Work engagement T1	0.650 (0.058)	<0.001	0.611 (0.066)	<0.001
Increasing challenging job demands T1	0.031 (0.052)	0.553	0.073 (0.059)	0.217
Decreasing hindering job demands T1	−0.074 (0.043)	0.084	−0.087 (0.046)	0.057
Increasing challenging job demands x Decreasing hindering job demands T1			0.139 (0.047)	0.003
D (Δdf)			8.89 (1)	0.003
Work engagement T1	0.647 (0.054)	<0.001	0.630 (0.057)	<0.001
Increasing social job resources T1	0.043 (0.045)	0.344	0.051 (0.048)	0.280
Decreasing hindering job demands T1	−0.080 (0.043)	0.064	−0.085 (0.044)	0.054
Increasing social job resources x Decreasing hindering job demands T1			0.106 (0.042)	0.012
D (Δdf)			6.40 (1)	0.011
Work engagement T1	0.695 (0.052)	<0.001	0.696 (0.053)	<0.001
Increasing structural job resources T1	−0.067 (.045)	0.135	−0.067 (0.045)	0.135
Decreasing hindering job demands T1	−0.068 (.042)	0.103	−0.068 (0.042)	0.107
Increasing structural job resources x Decreasing hindering job demands T1			−0.007 (0.046)	0.883
D (Δdf)			0.024 (1)	0.877

Note. M0 = model without interaction factor. M1 = model with interaction factor. β = standardized path coefficient. SE = standardized error. D = log-likelihood ratio test. df = degrees of freedom. T1 = Time 1; T2 = Time 2, 18 months after T1.

**Table 4:** Simple slopes between high decreasing hindering job demands and work engagement at high and low levels of increasing challenging job demands and increasing social job resources.

Interaction	Work Engagement T2 β (SE)	*p*
Increasing challenging job demands T1		
Low	−0.414 (0.171)	0.016
High	0.212 (0.098)	0.030
Increasing social job resources T1		
Low	−0.331 (0.143)	0.021
High	0.191 (0.102)	0.062

**Table 5:** Results of LMS: interaction effects of job crafting dimensions on work engagement among participants not facing changes in work content (*n* = 412).

T1 Variables	M0 T2 Work Engagement β (SE)	*p*	M1 T2 Work Engagement β (SE)	*p*
Work engagement T1	0.802 (0.043)	<0.001	0.803 (0.043)	<0.001
Increasing challenging job demands T1	−0.007 (0.050)	0.895	−0.010 (0.049)	0.840
Decreasing hindering job demands T1	0.035 (0.040)	0.391	0.031 (0.042)	0.451
Increasing challenging job demands x decreasing hindering job demands T1			0.030 (0.054)	0.580
D (Δdf)			0.522	0.819
Work engagement T1	0.787 (0.044)	<0.001	0.789 (0.044)	<0.001
Increasing social job resources T1	0.031 (0.045)	0.487	0.028 (0.045)	0.533
Decreasing hindering job demands T1	0.029 (0.041)	0.482	0.031 (0.041)	0.452
Increasing social job resources x decreasing hindering job demands T1			−0.066 (0.046)	0.148
D (Δdf)			2.33	0.127
Work engagement T1	0.807 (0.043)	<0.001	0.806 (0.043)	<0.001
Increasing structural job resources T1	−0.034 (0.044)	0.445	−0.036 (0.044)	0.409
Decreasing hindering job demands T1	0.031 (0.041)	0.448	0.030 (0.040)	0.460
Increasing structural job resources x decreasing hindering job demands T1			0.038 (0.045)	0.396
D (Δdf)			0.886	0.347

Note. M0 = model without interaction factor. M1 = model with interaction factor. β = standardized path coefficient. SE = standardized error. D = log-likelihood ratio test. df = degrees of freedom. T1 = Time 1; T2 = Time 2, 18 months after T1.

## Appendix A

The results from the full sample showed that job crafting at T1 had no significant main or interaction effects on work engagement at T2 after controlling for the level of work engagement at T1. More specifically, the interaction term of increasing challenging job demands and decreasing hindering job demands (β = 0.016, *p* = 0.471), the interaction term of increasing structural job resources and decreasing hindering job demands (β = 0.007, *p* = 0.698), and the interaction term of increasing social job resources and decreasing hindering job demands (β = 0.015, *p* = 0.440) for work engagement were all non-significant. Thus, the investigation of the effects of job crafting on work engagement as an average over the entire sample missed the heterogeneity of the dataset and neglected the different ways in which job crafting was related to work engagement among different groups of employees.
